# Plexiform fibromyxoma of the duodenum – specific imaging findings and pathological analysis of a rare site: A case report

**DOI:** 10.1097/MD.0000000000047048

**Published:** 2026-01-23

**Authors:** Guangliu Wu, Rujiao Yin, Kui Long

**Affiliations:** a Department of Hepatopancreatobiliary Surgery, The Second Affiliated Hospital of Kunming Medical University, Kunming, China; b Department of Radiology, The Second Affiliated Hospital of Kunming Medical University, Kunming, China.

**Keywords:** case report, CT, duodenum, mesenchymal tumors, MRI, plexiform fibromyxoma

## Abstract

**Rationale:**

Plexiform fibromyxoma (PF) is a rare, benign gastrointestinal mesenchymal tumor that rarely occurs in the duodenum and is easily confused with other tumors, leading to misdiagnosis. This article uses a rare duodenal case and combines imaging and pathological features to explore its diagnostic points to help improve the radiologists’ ability to recognize it.

**Patient concerns:**

A 51-year-old female patient was admitted to the hospital because of loss of appetite and fatigue for 1 week.

**Diagnoses:**

Imaging examination revealed a cystic solid mass in the duodenum, and enhanced scanning showed uneven progressive enhancement. The location and morphology of the mass changed during different examinations. Imaging diagnosis is considered a benign tumor of duodenal origin. The final diagnosis of duodenal PF was confirmed by histopathology and immunohistochemistry following surgical resection.

**Interventions:**

The patient underwent a radical pancreaticoduodenectomy.

**Outcomes:**

The patient had no intraoperative or postoperative complications and had no signs of recurrence during the 17-month and 5-year follow-up.

**Lessons:**

PF of the duodenum is a relatively rare tumor with specific imaging manifestations. Multiparameter magnetic resonance imaging examination can improve its diagnostic rate. We report a case of duodenal PF with complete CT and magnetic resonance imaging imaging examinations.

## 1. Introduction

Plexiform fibromyxoma (PF) is a rare, benign gastrointestinal mesenchymal tumor. It was first reported by Takahashi et al in 2007 and was named plexiform angiomyxoid myofibroblastic tumor, which emphasizes the presence of myofibroblast differentiation characteristics.^[1]^ {NOTE:Plexiform}In 2009, Miettinen et al classified it as PF, covering all previously described histological and immunohistochemical subtypes.^[2]^ In 2010, the WHO classification of digestive system tumors officially included it.^[3]^ PF mostly occurs in the gastric antrum but can also be found in the esophagus, duodenum, jejunum, gallbladder, and colon. The high incidence in the gastric antrum may be related to its tissue structure and cell composition. The antral muscle layer and submucosal layers are rich in smooth muscle and mesenchymal cells, and the antrum has a rich vascular network, which provides a favorable microenvironment for the occurrence of PF.^[4]^ It is extremely rare in the duodenum. Due to its atypical imaging manifestations and overlap with other intestinal mesenchymal tumors in imaging, it is easy to misdiagnose or miss in clinical practice. This report presents a rare case of duodenal PF and explores its imaging characteristics, pathological manifestations, and imaging differential diagnosis, aiming to improve the understanding and diagnostic sensitivity of imaging physicians for this disease.

## 2. Case presentation

**Figure 1.**
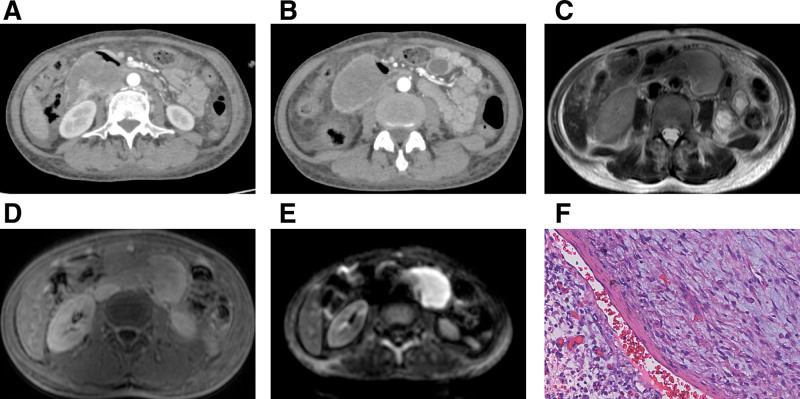
(A) The mass demonstrates mild heterogeneous enhancement during the arterial phase of contrast-enhanced CT, with a distinct enhanced nodule present. (B) Heterogeneous enhancement of the mass progresses during the delayed phase of contrast-enhanced CT, while the enhancement of the previously noted arterial phase nodule diminishes. (C) The mass located in the horizontal segment of the duodenum exhibits mixed, slightly hyperintense T2 signal intensity on T2-weighted MRI. (D) Moderate heterogeneous enhancement is observed during the delayed phase of MRI contrast administration. (E) The mass displays hyperintense signal intensity on diffusion-weighted imaging. (F) Histopathological examination confirms the presence of a PF (HE × 100). Image obtained on November 2024. Images courtesy of the authors (Rujiao Yin). CT = computed tomography, MRI = magnetic resonance imaging, PF = Plexiform fibromyxoma.

The patient was a 51-year-old female who was admitted to the hospital with a 1-week history of loss of appetite and fatigue. Laboratory tests revealed positive stool occult blood and a hemoglobin 51 g/L, indicating anemia. In this case, multidetector computed tomography (MDCT) and multiparametric magnetic resonance imaging (MRI) examinations were performed. CT was performed using an Aquilion ONE scanner with a slice thickness of 0.5 mm. Arterial phase (approximately 25–30 s), portal venous phase (approximately 60–70 s), and delayed phase (approximately 180 seconds) scans were obtained. The contrast agent used was iopamidol injection, administered at a rate of 4.0 mL/s with a total volume of 100 mL. MRI was performed using a Philips 3.0T system with a slice thickness of 4 to 8 mm, an inter-slice gap of 1 mm, FOV of 380 to 400 mm, and a matrix of 256 × 256. Sequences included T2-weighted axial, T2-weighted coronal, T1-weighted dual-echo (in/out-phase), diffusion-weighted imaging (b = 0 and 800 seconds/mm²), and 3D T1-weighted THRIVE (pre-contrast, arterial, portal venous, and delayed phases). The contrast agent used was Gd-DTPA (0.5 mmol/mL). Abdominal CT showed a cystic solid mass in the duodenum, about 5.1 × 5.2 × 5.0 cm in size. The CT values of the solid and cystic components of the mass were 36 HU and 14 HU, respectively. In the enhanced arterial phase, the CT values were 153 HU and 48 HU, and the CT values of the venous and delayed phase were 93 HU and 106 HU. The CT-enhanced arterial phase was slightly enhanced (Fig. 1A), and the delayed phase was progressively unevenly enhanced (Fig. 1B). Abdominal MRI showed a cystic solid mass of duodenal origin, T1WI showed a slightly low signal, T2WI showed an uneven slightly high signal (Fig. 1C), and after enhancement, it showed an uneven progressive enhancement (Fig. 1D), and DWI showed restricted diffusion (Fig. 1E). The imaging diagnosis considered a benign tumor of duodenal origin. The patient underwent a radical pancreaticoduodenectomy. During the operation, the tumor was located in the duodenal papilla, with a smooth surface, medium to soft texture, clear boundaries, and no invasion of the serosal layer or infiltration of adjacent organs. Postoperative pathology: a mass in the duodenal papilla, grayish white and tough. Under light microscopy, the tumor grew in a plexiform pattern, interlaced with smooth muscle tissue, with a stroma rich in myxoid matrix and thin-walled small blood vessels, with inconspicuous nucleoli and rare nuclear divisions. Immunohistochemistry: CD34 (vessel +), VIM (+), CD138 (+), CD163 (+), Fli-1 (+), SDHB (+), DOG-1 (±), SMA (+), Ki-67 (5%), CD34 (+), β-catenin (±). Pathological diagnosis: PF of the duodenum (Fig. 1F).

## 3. Discussion

PF is a rare, benign mesenchymal tumor of the gastrointestinal tract.^[5]^ Previous reports have shown no significant gender difference in the incidence of PF. Clinically, it is mostly caused by compression of the tumor or submucosal infiltration leading to mucosal ulcers, causing upper gastrointestinal bleeding, anemia, obstruction, or nonspecific upper abdominal discomfort. The main manifestations of this patient were anemia and gastrointestinal symptoms, indicating tumor compression and mucosal destruction, with typical rarity and clinical characteristics. Currently, the preferred treatment for PF is surgical resection, and the patient has a good prognosis. There have been no reports of recurrence and metastasis at home or abroad.^[6]^ The patient’s hemoglobin was 115 g/L 6 months after surgery, and the anemia was corrected. No local recurrence or distant metastasis was found on follow-up imaging, which is consistent with the literature reports.

Duodenal PF is a relatively rare tumor with specific imaging manifestations. MRI with multiple parameters can improve its diagnostic rate. Pathological and imaging features: The duodenal cavity was cystically located between the intestinal submucosal fibrous tissue and smooth muscle. Simultaneously, because CT and MRI were performed at different times, the location and morphology of the lesion were inconsistent. It deformed with the change in intestinal morphology, indicating that the lesion was relatively soft. There were no previous literature reports on this feature, so we were lucky. We hope that reporting this imaging feature will help to differentiate it from other duodenal tumors. CT plain scan showed a cystic and solid mass. During the arterial phase of CT enhancement, obvious enhancement areas were visible in the mass, and the enhancement slightly decreased in the later period. Combined with pathology, it is speculated that this is related to the large number of thin-walled capillaries in the tumor. The cystic part of the mass on the CT plain scan showed watery density, and the MRI plain scan T2WI image showed a high signal. Dynamic enhancement showed progressive delayed enhancement, which was consistent with previous literature reports.^[7]^ Combined with pathology, it is speculated that this is mainly related to the presence of fiber and mucin matrix components in the tumor, and the more mucin components, the softer the tumor. Diffusion-weighted imaging showed a high signal, and combined with pathology, it was speculated that the tumor clustered and the mucoid matrix contained a large amount of acidic mucopolysaccharides, which led to restricted movement of water molecules and thus limited diffusion. This case has specific imaging manifestations: the location and morphology of the lesion change with the changes in the intestinal morphology, the intestinal tube is dilated but the intestinal wall is not thick, the CT plain scan shows water-like density, and the CT and MRI dynamic enhancement show progressive delayed enhancement. If the lesion is small or only noncontrast CT is performed, it is often easily missed. The diagnosis of this tumor requires dynamic contrast-enhanced CT and multisequence MRI examinations. Dynamic contrast-enhanced CT can clearly demonstrate the cystic and solid components of the mass as well as its vascular enhancement characteristics. Multisequence and dynamic contrast-enhanced MRI (including high T2 signal intensity, progressive delayed enhancement, and DWI/ADC features) are helpful in differentiating mucin-rich lesions from other stromal tumors. This tumor also needs to be differentiated from stromal tumors, inflammatory fibrous polyps, inflammatory myofibroblastic tumors, neurothecomas, and glomus tumors. Duodenal stromal tumor: Often exophytic or mixed growth, tumors are mostly located outside the intestinal cavity, CT and MRI plain scans show soft tissue density/signal masses, and after enhancement, they mostly exhibit nonuniform delayed enhancement. Cystic changes and necrosis are common, and they are often accompanied by intestinal wall thickening. Inflammatory fibrous polyp: A rare, benign gastrointestinal stromal lesion, usually originating from the submucosal layer. It is most common in the gastric antrum, rare in the duodenum, mostly solid, with mild to moderate uniform enhancement, and lacks cystic changes and delayed enhancement. Inflammatory myofibroblastic tumor: It is extremely rare in the duodenum. CT and MRI plain scans show mixed density/signal, and after enhancement, it demonstrates nonuniform progressive enhancement. The degree of enhancement is related to the components in the tumor, and the delayed enhancement is faster than that of PF; Neurofibroma: T2WI image has high signal, often with a “target sign,” nonuniform progressive enhancement, or ring enhancement. Glomus tumor: A solid mass with delayed uniform enhancement.

Comprehensive clinical, imaging, and pathological results, along with the rare occurrence site and specific imaging manifestations of PF in this case, emphasize the importance of understanding and differential diagnosis of this disease. Complete surgical resection is the only effective treatment. Postoperative follow-up showed good prognosis without recurrence or metastasis.

## Author contributions

**Validation:** Kui Long.

**Writing – original draft:** Guangliu Wu.

**Writing – review & editing:** Rujiao Yin.
